# Reserving Interior Void Space for Volume Change Accommodation: An Example of Cable‐Like MWNTs@SnO_2_@C Composite for Superior Lithium and Sodium Storage

**DOI:** 10.1002/advs.201500097

**Published:** 2015-05-15

**Authors:** Yi Zhao, Chao Wei, Shengnan Sun, Luyuan Paul Wang, Zhichuan J. Xu

**Affiliations:** ^1^ School of Materials Science and EngineeringNanyang Technological UniversitySingapore639798Singapore; ^2^ Energy Research Institute @ NTUNanyang Technological University50 Nanyang Drive, SingaporeSingapore639798Singapore

**Keywords:** carbon layer, carbon nanotubes, lithium‐ion batteries, SnO_2_ anode, sodium‐ion batteries

## Abstract

**Reserving interior void space in the cable‐like structure of multiwalled carbon nanotubes‐in‐SnO_2_‐in‐carbon layer (MWNTs@SnO_2_@C)** is reported for the first time. Such a design enables the structure performing excellent for Li and Na storage, which benefit from the good electrical conductivity of MWNTs and carbon layer as well as the reserved void space to accommodate the volume changes of SnO_2_.

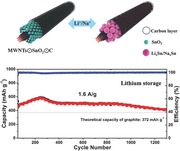

For reducing carbon emission and fossil fuel consumption, lithium‐ion batteries (LIBs) are now receiving a great deal of attention for transportation systems (e.g., hybrid electric vehicles and electric vehicles) and stationary storage of intermittent renewable energies such as solar and wind. To meet the increasing demand, it is highly desired to have next generation LIBs with higher power and energy densities, longer cycling life, improved safety, and lower cost.^1^ In the past decade, transition metal oxides with high capacities from 600 to 1200 mAh g^−1^ have been widely investigated as alternative anode materials to the commercial graphite anode (theoretical capacity of 372 mAh g^−1^).^2^ Among them, SnO_2_ has received particular attention due to its high theoretical capacity of 1493 mAh g^−1^ and the safe working potential. However, due to the low electrical conductivity and large volume change (>300%) during lithium insertion and extraction processes, SnO_2_ anode suffered from slow kinetics of charge diffusion as well as the agglomeration and pulverization problems during cycles, which resulted in the poor electrochemical performance with fast capacity fading and low rate capability.

To improve the electrochemical performance of SnO_2_ anode, two strategies have been proposed and employed. One is nanostructured SnO_2_ with various morphologies,^3^ which could provide large electrode/electrolyte contact area, abundant active sites for Li storage, and short diffusion path for electron and ions. Thus, the nanosized SnO_2_ was able to show enhanced performance compared to their bulk counterparts. For instance, SnO_2_ nanosheets can deliver a high capacity of 980 mAh g^−1^ in the initial charge cycle with the retention of 57% after 20 cycles.^4^ However, the pulverization problem for nanostructured SnO_2_ still persisted after several cycles, resulting in poor cycling stability with gradual capacity fading.

The other strategy is to hybrid SnO_2_ with carbon nano­materials (e.g., carbon coating layers, porous carbons, carbon nanotubes/nanohorns, and graphene),^5^ which could enhance the electrical conductivity by SnO_2_–carbon composite and alleviate the volume expansion/shrinkage to maintain the structure stable. Carbon coating has been widely used to improve the electrochemical performance of metal oxide anodes for LIBs. For example, Lou et al. found that SnO_2_@C microboxes exhibited better electrochemical performance (550 mAh g^−1^ remained after 150 cycles at 200 mA g^−1^) than the pure SnO_2_ microboxes without coating.^6^ However, it should be noticed that the carbon coating layer usually is given by amorphous carbon, which is rich in structure defects and would give the electrical conductivity much lower than the expected. As a result, the rate performance is limited.^7^ To further improve the electrical conductivity, carbon nanotubes (CNTs) and graphene were employed to support SnO_2_. These composites showed high specific capacity and good rate capability. However, due to the large volume changes, the aggregation of SnO_2_ particles (especially for high loading ratios) on CNTs or graphene was inevitable, thus damaging the structure stability and lithium storage performance.^8^ To mitigate these problems, SnO_2_ attached on CNTs and graphene were further coated with a conductive protection layer.^9^ For example, CNTs@SnO_2_ with a carbon coating layer (coaxial nanocables) reported by Yang et al. exhibited better cycling stability than the one without coating.[[qv: 9b]] However, another issue has not been completely solved is the volume change of SnO_2_. Due to the lack of void space in these composites, the carbon coating layers suffered from severe volume variation of SnO_2_ during lithium insertion/extraction and eventually can be destroyed after long‐time cycles. Therefore, the fabrication of SnO_2_ confined within hybrid carbon structures with void space is essential to achieve excellent and long‐life lithium storage performance.^10^

Recently, Na‐ion batteries (NIBs) have attracted increasing attention as an alternative to LIBs for large‐scale energy storage systems, owing to their low cost and the abundant sodium resources.^11^ Compared to Li^+^ ion (0.59 Å), Na^+^ ion (1.02 Å) has a larger ionic radius, resulting in a low sodium ion diffusion coefficient and poor insertion property into graphite.^12^ To develop suitable anode materials, transition metal oxides with high theoretical sodium storage capacity have been also investigated for NIBs.^13^ Among them, SnO_2_ as an anode material for NIBs delivered a theoretical capacity of 667 mAh g^−1^ by reversible forming a Na_15_Sn_4_ alloy.^14^ Similar with LIBs, combining SnO_2_ with carbonaceous materials has been adopted to improve its sodium storage performance.^15^ For example, Wang's group reported a SnO_2_/graphene composite with stable discharge capacity of 302 mAh g^−1^ at 160 mA g^−1^ after 100 cycles.[[qv: 15b]] However, the conversion from Sn to Na_15_Sn_4_ alloy suffered from an extremely large volume change of 420%, which is higher than that (250%) from Sn to Li_22_Sn_5_.^16^ In this regard, large void space is more needed for SnO_2_ composites to obtain high performance NIBs anode materials.

To achieve the above functions, here we present a rational design and synthesis of a novel nanostructure of multiwalled carbon nanotubes (MWNTs)‐in‐SnO_2_‐in‐carbon layer (MWNTs@SnO_2_@C) composite for high performance LIBs and NIBs. Seen from the schematic illustration in **Figure** **1**, MWNTs were sequentially coated with thick SiO_2_, SnO_2_, thin SiO_2_, and carbon layers. After the removal of SiO_2_ layers, the final MWNTs@SnO_2_@C composite with large internal void space was obtained. This unique architecture was able to enhance the electrical conductivity, facilitate the formation of stable solid electrolyte interface (SEI) layer, prevent the aggregation of SnO_2_, and provide large void space to accommodate the volume changes of SnO_2_ during cycles. As a result, the MWNTs@SnO_2_@C composite as anode materials for LIBs and NIBs delivered outstanding electrochemical performance with high specific capacity, good rate performance as well as excellent cycling stability.

**Figure 1:**
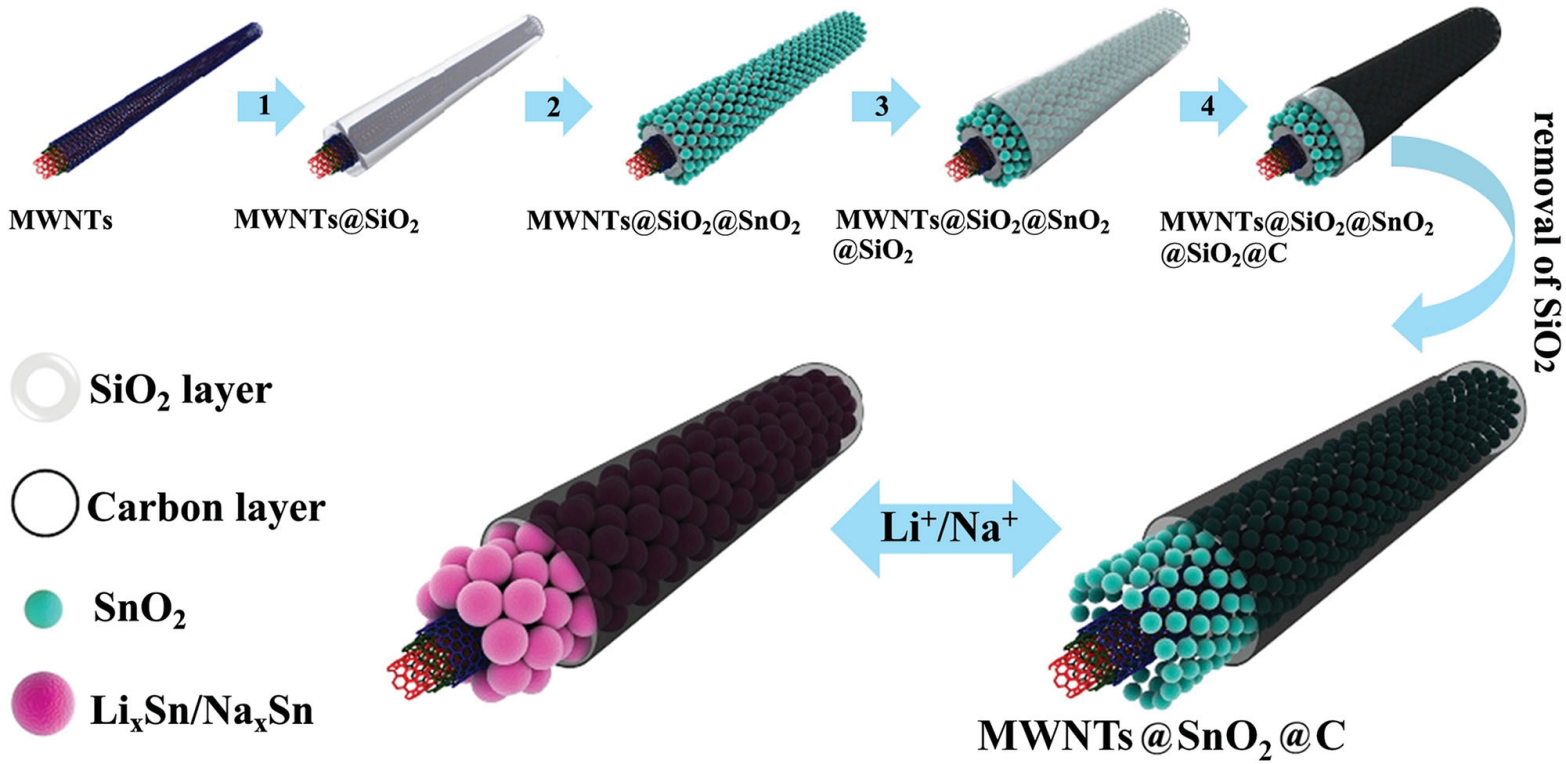
Schematic illustration for the fabrication of MWNTs@SnO_2_@C composite. First, MWNTs were sequentially coated with thick SiO_2_, SnO_2_, thin SiO_2_, and carbon layers from steps 1 to 4. After the removal of SiO_2_ layers, an MWNTs@SnO_2_@C composite with large internal void space can be finally obtained, which could effectively accommodate the volume changes of SnO_2_ during Li^+^ or Na^+^ insertion/extraction cycles.

The morphology and structure of the obtained composites were characterized by scanning electron microscope (SEM) and transmission electron microscope (TEM) in **Figure** **2**. As can be seen, the MWNTs@SiO_2_, MWNTs@SiO_2_@SnO_2_, and MWNTs@SiO_2_@SnO_2_@SiO_2_@C composites all remained the 1D morphology of MWNTs with increasing dia­meters.^17^ No other particles were observed outside these composites, indicating the uniform layer coatings during the whole synthesis process. MWNTs were first coated with thick SiO_2_ layers via a modified Stöber method. The obtained MWNTs@SiO_2_ composite had a thick SiO_2_ layer around 40 nm (Figure 2c), which can provide large internal void space in the final composite. Figure 2d–f displays the SEM and TEM images of MWNTs@SiO_2_@SnO_2_ composite achieved by the hydrothermal hydrolysis of K_2_SnO_3_ on SiO_2_ layer.[[qv: 5b]] To increase the loading ratio of SnO_2_, this hydrothermal process was repeated three times. The TEM image in Figure 2f disclosed the existence of three SnO_2_ layers, which were consisted with 2–3 nm SnO_2_ nanoparticles (Figure S1, Supporting Information). Then, a thin SiO_2_ layer was further applied on MWNTs@SiO_2_@SnO_2_ composite (Figure S2, Supporting Information), to facilitate the coating of resorcinol–formaldehyde (RF) resin derived carbon layer, which had advantages of low cost, good electrical conductivity, and outstanding thermal and mechanical properties.^18^ The RF resin coating on SiO_2_ was realized via a sol–gel process reported by Yin's group.^19^ After carbonization at 700 °C under Ar atmosphere, the RF‐derived carbon layer could exhibit good electrical conductivity, and the intermediate thin SiO_2_ was able to prevent the SnO_2_ layer from being reduced by carbon. Seen from the TEM image of MWNTs@SiO_2_@SnO_2_@SiO_2_@C composite in Figure 2i, the particle size of SnO_2_ increased up to 8–12 nm after carbonization. And the coating thickness outside SnO_2_ layer was about 13–18 nm, which was larger than the thickness of thin SiO_2_ layer (≈8 nm) in Figure S2 (Supporting Information), indicating the existence of carbon coating in this composite.

**Figure 2:**
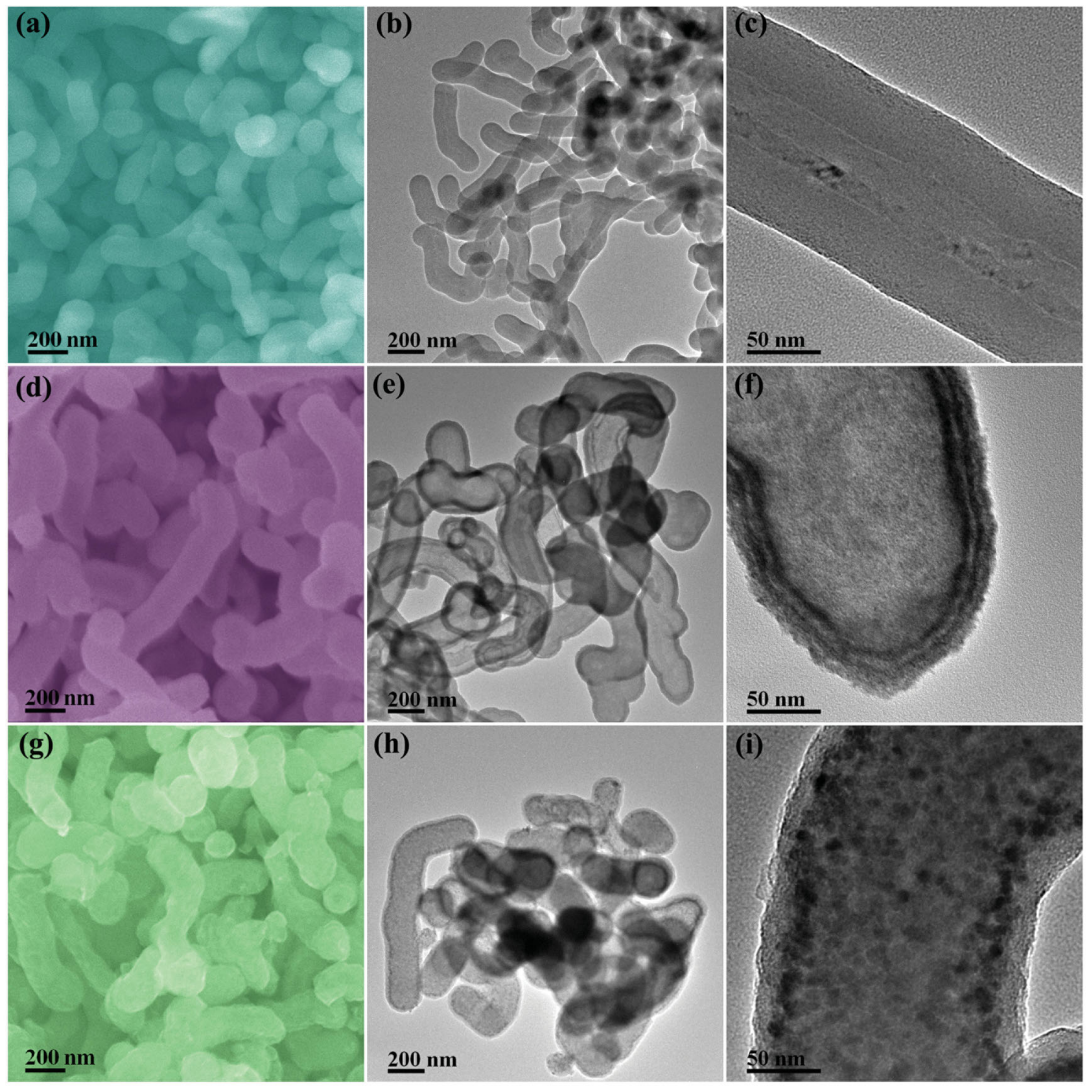
SEM and TEM images of a–c) MWNTs@SiO_2_, d–f) MWNTs@SiO_2_@SnO_2_, and g–i) MWNTs@SiO_2_@SnO_2_@SiO_2_@C composites.

**Figure** **3** displays the SEM and TEM images of the final obtained MWNTs@SnO_2_@C composite, which also presents 1D morphology with diameters around 150–250 nm and several micrometers in length. After the removal of SiO_2_ layers, SnO_2_ nanoparticles encapsulated within carbon layers with internal void space was obviously observed (Figure 3a–e). According to the TEM image in Figure 3e, the thickness of the carbon layer was around 6–10 nm. Figure 3f evidently revealed the existence of MWNTs inside this composite. Meanwhile, the high‐resolution TEM (HRTEM) image (inset in Figure 3f) of SnO_2_ nanoparticle exhibited a regular interlayer spacing of 0.33 nm, which was ascribed to the (110) planes of tetragonal SnO_2_. To further illustrate the spatial distribution of SnO_2_, carbon layer, and MWNTs in MWNTs@SnO_2_@C composite, element mappings for carbon, tin, and oxygen were carried out. The existence of MWNTs inside this composite was further demonstrated by the carbon mapping in Figure 3h. The results in Figure 3h–j demonstrated that SnO_2_ layer was uniformly distributed between MWNTs and carbon coating with large interior void space. As a comparison, the MWNTs@SnO_2_ composite was also synthesized by directly etching the MWNTs@SiO_2_@SnO_2_ composite with NaOH solution, from which MWNTs confined within hollow SnO_2_ nanotubes with large empty space was observed in Figure S3 (Supporting Information).

**Figure 3:**
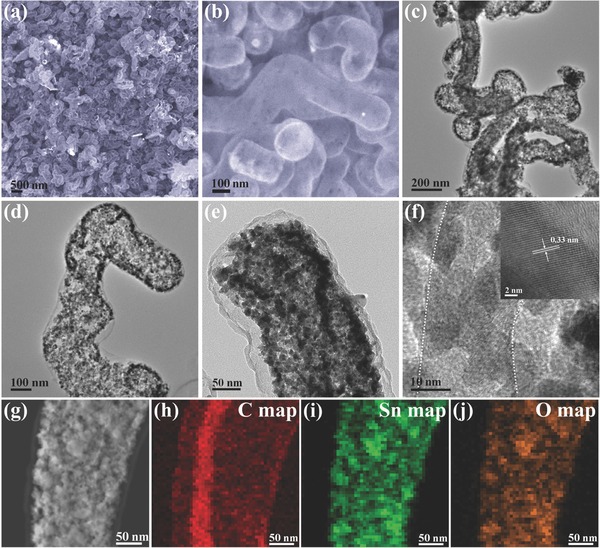
a,b) SEM and c–f) TEM images of the MWNTs@SnO_2_@C composite. Inset in Figure 3f is the HRTEM image of SnO_2_ nanoparticle. g) Scanning transmission electron microscopy image and the corresponding element mapping images of h) carbon, i) tin, and j) oxygen for MWNTs@SnO_2_@C composite, demonstrating the well confined of SnO_2_ nanoparticles between MWNTs and carbon layers.

The crystalline structure of MWNTs@SnO_2_@C composites was characterized by X‐ray diffraction (XRD) in **Figure** **4**a. As can be seen, the main diffraction peaks of this composite can be well assigned to the tetragonal structure of SnO_2_ (JCPDS No. 41‐1445). The diffraction peaks in MWNTs@SnO_2_@C composite were much sharper than the MWNTs@SnO_2_ composite (Figure S4, Supporting Information), indicating the larger SnO_2_ particles size after carbonization, in consistence with the TEM observation. The diffraction peak from MWNTs near 26° was overlapped with the (110) planes of SnO_2_. The weight content of SnO_2_ was determined by the thermogravimetry analysis from 30 to 700 °C with a heating rate of 10 K min^−1^ in air. As shown in Figure S5 (Supporting Information), the loading ratio of SnO_2_ in MWNTs@SnO_2_@C composite is about 57 wt%. Two distinct weight loss regions at 400–580 and 580–670 °C were observed in the thermogravimetry analyses (TGA) curve, corresponding to the removal of carbon layer and MWNTs, respectively.[[qv: 9b]] To further investigate the structure properties of MWNTs@SnO_2_@C composite, nitrogen adsorption–desorption isotherms was performed in Figure 4b. The specific Brunauer–Emmett–Teller (BET) surface area and total pore volume of the MWNTs@SnO_2_@C composite were 218 m^2^ g^−1^ and 0.33 cm^3^ g^−1^, respectively. Seen from the pore size distribution curve (inset in Figure 4b), this composite owns mesopores at 2.0 and 3.8 nm as well as a broad pore distribution from 4.3 to 100 nm, which could be resulted from the mesopores between SnO_2_ nanoparticles and the removal of SiO_2_ layers. The large surface area and pore volume in this composite could increase the electrode/electrolyte contact area for fast charge transportation and provide void space to accommodate the volume changes of SnO_2_ during cycles, which is essential to achieve excellent electrochemical performance as anode materials for LIBs and NIBs.

**Figure 4:**
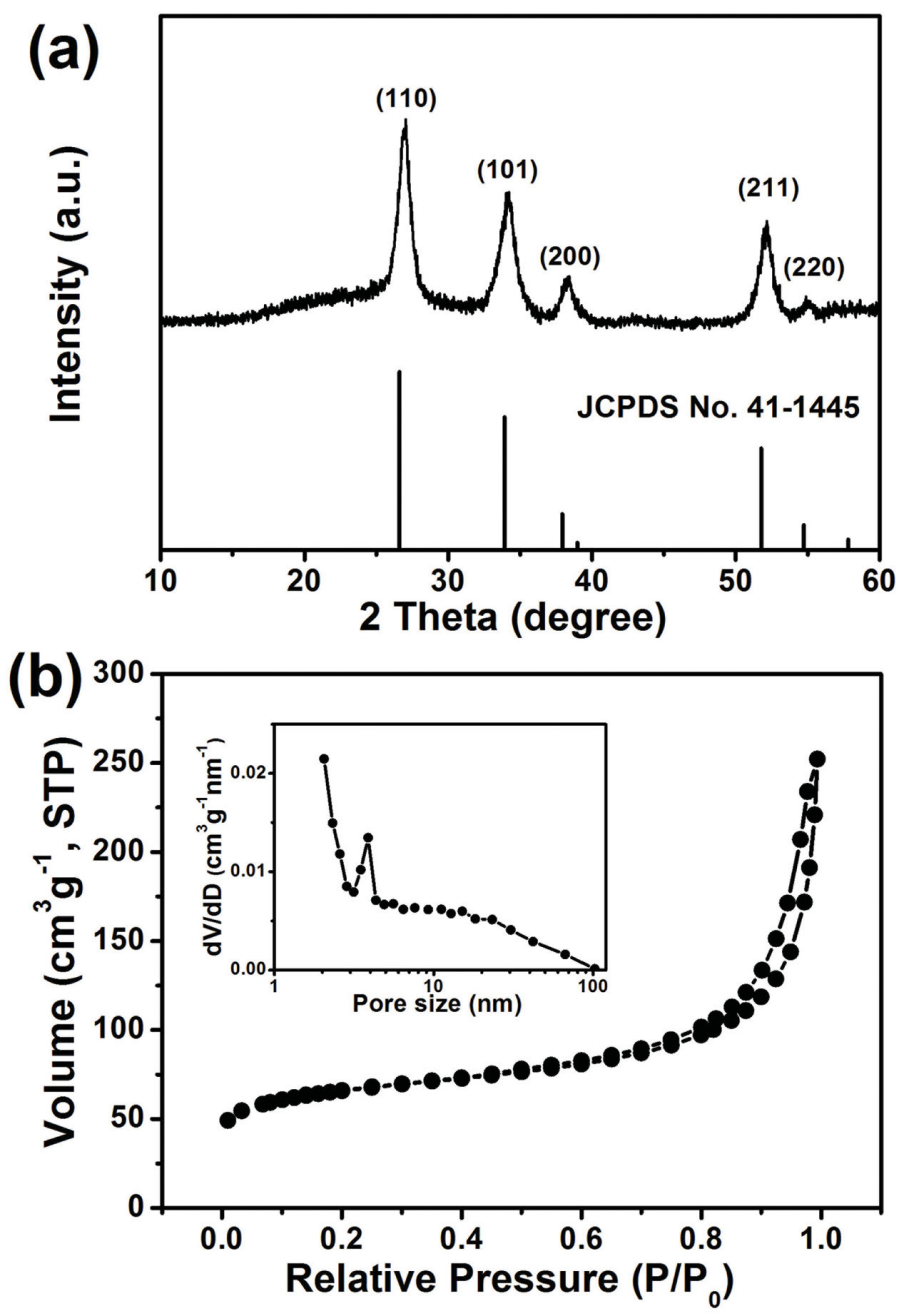
a) XRD pattern and b) nitrogen adsorption–desorption isotherms of MWNTs@SnO_2_@C composite. Inset in (b) is the corresponding pore size distribution.

**Figure** **5** reveals the electrochemical performance of the MWNTs@SnO_2_@C composite as an anode material for LIBs. The cyclic voltammograms (CVs) were first carried out to investigate the electrochemical behavior of MWNTs@SnO_2_@C electrode. Figure 5a exhibited the initial three CV curves of the electrode at a scan rate of 0.5 mV s^−1^ between 0.05 and 3.00 V. A broad reduction peak around 0.8 V was observed in the first cathodic scan, which was attributed to the formation of SEI layer and the conversion of SnO_2_ to Sn in Reaction (1). In the second cycle, this peak disappeared and only two small peaks around 0.9 and 1.1 V corresponded to conversion reaction were observed, indicating the formation of a stable SEI layer after the first cycle. The anodic peaks at 1.3 and 1.9 V were ascribed to the reverse processes from Sn to SnO_2_. Meanwhile, a pronounced redox pairs located at around (cathodic/anodic) 0.05/0.6 V was clearly detected in the CVs, corresponding to the highly reversible alloying and dealloying processes of Li*_x_*Sn in Reaction (2).[[qv: 9d]],^20^

(1) $${\mathrm{SnO}}_{2}+4{\mathrm{Li}}^{+}+4e^{-}↔\mathrm{Sn}+2{\mathrm{Li}}_{2}O$$

(2) $$x{\mathrm{Li}}^{+}+\mathrm{Sn}+{\mathrm{xe}}^{-}↔{\mathrm{Li}}_{x}\mathrm{Sn}\;\left(0\leq x\leq 4.4\right)$$

**Figure 5:**
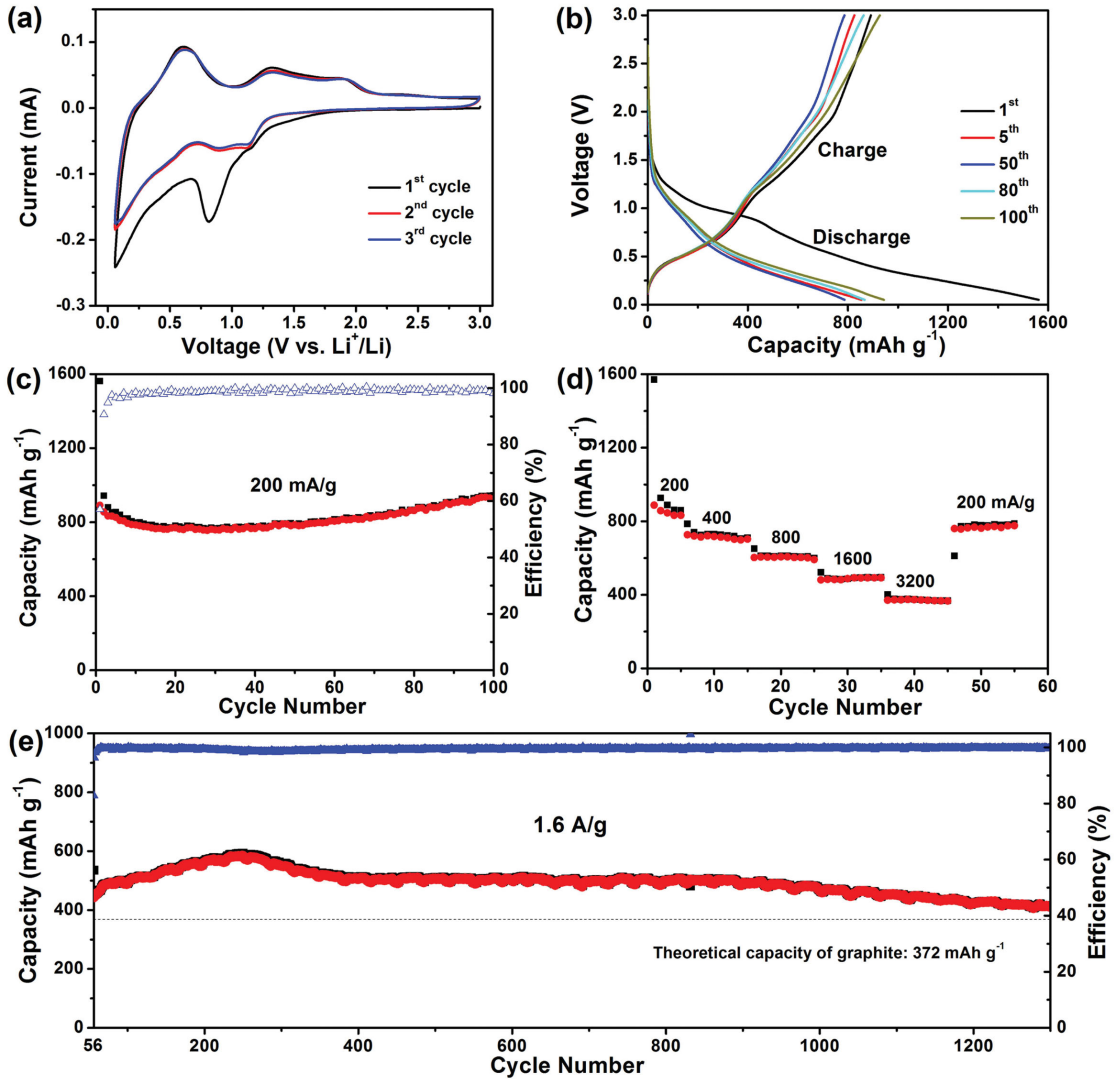
Electrochemical performance of MWNTs@SnO_2_@C anode for LIBs. a) CV curves of the first three cycles at a scan rate of 0.5 mV s^−1^ under a voltage range of 0.05–3.00 V. b) Discharge/charge curves for selected cycles and c) cycling performance and columbic efficiencies at 200 mA g^−1^. d) Rate capabilities at various current densities from 200 to 3200 mA g^−1^. d) Long‐term cycling stability at a high rate of 1.6 A g^−1^ up to 1300 cycles.

The cycling stability and lithium storage performance of the MWNTs@SnO_2_@C electrode were studied by galvanostatic discharge–charge method at a current density of 200 mA g^−1^ from 0.05 to 3.00 V. As shown in Figure 5b, sloping plateau ascribed to the conversion and alloy reactions for SnO_2_ were observed in the first discharge/charge profiles, in accordance with the CV observation. Moreover, these plateaus were still found in the following cycles, indicating the stable electrochemical process during cycles. The discharge and charge capacities in the first cycle were 1562 and 892 mAh g^−1^, respectively, with a columbic efficiency (CE) of 57.1%. This initial capacity loss was generally resulted from the irreversible formation of the SEI layer and electrolyte decomposition. While, this value was higher than the initial CE of 52.2% for MWNTs@SnO_2_ anode (Figure S7, Supporting Information), which could be ascribed to the existence of carbon layer, to facilitate the formation of stable SEI layer and prevent the loss of active materials during cycle.^21^ Moreover, the CE for MWNTs@SnO_2_@C electrode quickly increased to around 98–99% in the following cycles. Figure 5c disclosed the high capacity and good cycling performance of MWNTs@SnO_2_@C electrode. After 100 successive discharge/charge cycles, the MWNTs@SnO_2_@C composite still delivered a high discharge capacity of 944 mAh g^−1^, which was superior than most of the reported SnO_2_ based composites. Meanwhile, an increase of capacity during the later cycles was noted, which could be resulted from the well confined of SnO_2_ NPs within MWNTs and carbon coating during cycles, to prevent the mass loss and facilitate the reversible conversion between SnO_2_ and Sn with increased capacity. Besides, the reversible formation of polymeric gel‐like film from the decomposition of electrolyte as well as the interfacial storage of excess lithium ions between the primary nanoparticles, which have been found in many metal oxides anode materials, might also contribute to such high specific capacity of this composite.[[qv: 5g]],[[qv: 9e]],^20^,22 In contrast, the MWNTs@SnO_2_ composite, which also had void space (Figure S6, Supporting Information) but without carbon layer protection, suffered from gradual capacity fading with only 527 mAh g^−1^ remained after 100 cycles (Figure S7, Supporting Information).

Figure 5d illustrates the excellent rate capabilities of MWNTs@SnO_2_@C anode at various current densities from 200 to 3200 mA g^−1^. The typical discharge and charge profiles at different rates were showed in Figure S8 (Supporting Information), which kept similar curve shape with high specific capacities even at high rates. The electrode remained stable charge capacities of 720, 606, 492, and 375 mAh g^−1^ when cycled at 400, 800, 1600, and 3200 mA g^−1^, respectively. Moreover, a high capacity of 770 mAh g^−1^ can be restored when back to 200 mA g^−1^, indicating the good structure stability of the MWNTs@SnO_2_@C composite. While, MWNTs@SnO_2_ electrode only delivered a low charge capacity of 250 mAh g^−1^ at 3200 mA g^−1^ (Figures S7 and S9, Supporting Information). Compared with the recent reported hollowed SnO_2_‐in‐TiO_2_ wire‐in‐tube composite,[[qv: 10c]] our composite exhibited better rate capabilities due to the existence of MWNTs and carbon layers with good electrical conductivity.

This electrode was also cycled at large current densities for long‐term cycling test. Figure 5e demonstrates the excellent cycling stability of the MWNTs@SnO_2_@C electrode at a high rate of 1.6 A g^−1^ after various discharge/charge rates test in Figure 5d. The electrode was able to maintain high discharge capacities of 502 and 412 mAh g^−1^ after 800 and 1300 cycles, respectively. The average capacity loss was only 0.025 mAh g^−1^ per cycle. Meanwhile, the CEs during the cycles kept above 99%, showing the good electrochemical reversibility of active materials. Besides, this MWNTs@SnO_2_@C electrode also delivered outstanding cycling performance at a high current density of 1 A g^−1^, which preserved a high discharge capacity of 513 mAh g^−1^ after 800 cycles (Figure S10, Supporting Information). On the contrary, the MWNTs@SnO_2_ electrode showed a very poor cycling stability at 1 A g^−1^ and only remained a low capacity of 96 mAh g^−1^ after 500 cycles (Figure S11, Supporting Information). Compared with the reported CNTs/graphene@SnO_2_ and CNTs/graphene@SnO_2_@C,[[qv: 5c]],[[qv: 9b]],c,e,^23^ the MWNTs@SnO_2_@C composite delivered significantly improved cycling stability due to its large interior void space to accommodate volume changes of SnO_2_ during cycles (Table S1, Supporting Information). To the best of our knowledge, the outstanding electrochemical performance of MWNTs@SnO_2_@C electrode with high reversible capacity (944 mAh g^−1^ remained after 100 cycles at 200 mA g^−1^) and excellent cycling stability (412 mAh g^−1^ after 1300 cycles at 1.6 A g^−1^) is one of the best results for SnO_2_ based anodes in LIBs.

To further evaluate the structure advantage of MWNTs@SnO_2_@C composite for sodium storage, the electrochemical performance of this composite as an anode material for NIBs was also characterized. **Figure** **6**a shows the initial three CV curves of the electrode with a scan rate of 0.5 mV s^−1^. Similar with the CVs for LIBs, the first cathodic scan exhibited a broad peak around 1.0 V, which was ascribed to the formation of SEI layer and the reduction of SnO_2_ to Sn in Reaction (3). This peak disappeared in the following cycles, indicating the irreversible property of these two reactions. The reduction peaks in the region from 0.66 to 0.05 V were ascribed to the formation of series of Na*_x_*Sn alloy in Reaction (4). The oxidation peaks around 1.2–1.5 V in the anodic process corresponded to the reversible dealloying of Na*_x_*Sn.^14^, ^15^,[qv: 15d]]

(3) $${\mathrm{SnO}}_{2}+4{\mathrm{Na}}^{+}+4e^{-}\to \mathrm{Sn}+2{\mathrm{Na}}_{2}O$$

(4) $$x{\mathrm{Na}}^{+}+\mathrm{Sn}+{\mathrm{xe}}^{-}↔{\mathrm{Na}}_{x}\mathrm{Sn}\;\left(0\leq x\leq 3.75\right)$$

**Figure 6:**
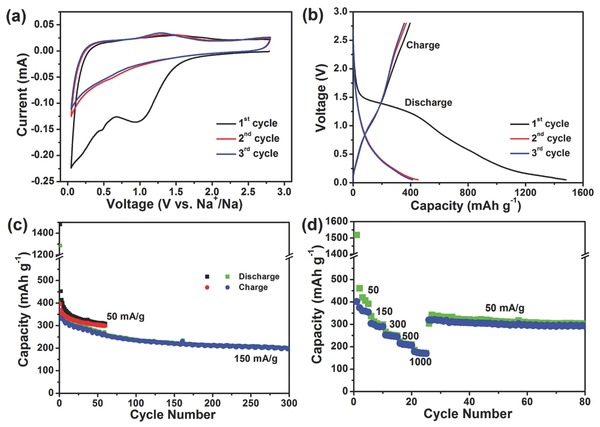
Electrochemical performance of MWNTs⊙SnO_2_⊙C anode for NIBs. a) CV curves at 0.5 mV s^−1^ between 0.05 and 2.80 V, b) discharge/charge profiles for the initial three cycles at 50 mA g^−1^, c) cycling performance at 50 and 150 mA g^−1^, and d) rate capabilities at various current densities from 50 to 1000 mA g^−1^.

Seen from the discharge/charge profiles cycled at 50 mA g^−1^ in Figure 6b, the discharge capacity in the first cycle was 1481 mAh g^−1^ and dropped to 452 mAh g^−1^ in the second cycle. This large capacity difference between the initial two cycles were mainly attributed to the formation of SEI layer and irreversible conversion between SnO_2_ and Sn, in consistence with the CVs result. Figure 6c disclosed the good cycling performance of the MWNTs@SnO_2_@C electrode for sodium storage. At 50 mA g^−1^, the charge capacity still remained at 300 mAh g^−1^ with capacity retention of 76% over 60 cycles. Moreover, an excellent long‐term cycling performance was also observed at 150 mA g^−1^ with a capacity of 200 mAh g^−1^ retained after 300 cycles. The MWNTs@SnO_2_@C electrode also displayed excellent rate capabilities for NIBs. As shown in Figure 6d and the typical discharge/charge profiles in Figure S12 (Supporting Information), this electrode delivered discharge capacities of 420, 313, 255, 213, and 176 mAh g^−1^ when cycled at 50, 150, 300, 500, and 1000 mA g^−1^, respectively. When the current density returned back to 50 mA g^−1^, the discharge capacity can be recovered to 337 mAh g^−1^ and remained stable up to 80 cycles, indicating that the MWNTs@SnO_2_@C composite can accommodate high‐rate sodium insertion/extraction cycling with good structural integrity. The above results demonstrated that the unique structure of MWNTs⊙SnO_2_⊙C composite also benefit for superior sodium storage performance in NIB.

To investigate the structure stability of MWNTs@SnO_2_@C composite during lithium and sodium storage process, we decomposed the cells after discharge/charge cycles and characterized the morphology by SEM. As shown in Figure S13 (Supporting Information), the MWNTs@SnO_2_@C electrodes were able to preserve the original 1D morphology even after several lithium or sodium insertion/extraction cycles, demonstrating the good structure stability of this unique architecture during cycles. While, the MWNTs@SnO_2_ electrode suffered from structure pulverization after cycles. Thus, the remarkable electrochemical performance of the MWNTs@SnO_2_@C composite could be attributed to the following factors. First, the 1D MWNTs and carbon coating greatly improved the electrical conductivity of this composite. Meanwhile, the nanosized SnO_2_ provided short lithium and sodium ions diffusion path, which were both beneficial for the good rate capabilities. Second, the carbon layer facilitated the formation of stable SEI layer, and prevented the aggregation and mass loss of SnO_2_ during cycles. Third, the large void space in this composite can effectively accommodate the volume changes of SnO_2_ during lithium and sodium insertion/extraction cycles with good structure stability, resulting in high specific capacity and outstanding cycling performance.

In conclusion, we have successfully designed and synthesized a unique MWNTs@SnO_2_@C composite, in which SnO_2_ NPs were confined within MWNTs and carbon layers with large interior void space. Such a structure design, benefiting from the good electrical conductivity of MWNTs and carbon layers as well as the large void space to accommodate the volume changes of SnO_2_ during cycles, was able to deliver outstanding lithium and sodium storage performance with high reversible capacity, excellent rate performance as well as remarkable cycling stability. In addition, this design also offers a general strategy to accommodate other electrode materials, such as Si, Sn, metal oxides, and sulfur, which give large volume changes during lithium or sodium insertion/extraction, to achieve good cycling and rate performance.

## Experimental Section

*Synthesis of MWNTs@SiO_2_ Composite*: MWNTs with diameter around 20–50 nm (purchased from Shenzhen Nanotech Port Co. Ltd) were first refluxed in nitric acid (65 wt%) at 140 °C for 6 h, denoted as acid‐MWNTs. 20 mg acid‐MWNTs was dispersed into a solution containing 71.4 mL ethanol, 10 mL deionized water, and 3.14 mL NH_3_·H_2_O (25–28 wt%) and sonicated for 3 h to form a homogeneous solution. Then, a mixture of 0.68 mL tetraethoxysilane (TEOS) and 5 mL ethanol was dropped into the above solution and stirred for 3 h. The obtained composite was filtered, washed with water and ethanol, and dried at 80 °C overnight.

*Synthesis of MWNTs@SiO_2_@SnO_2_ Composite*: The SnO_2_ coating was prepared based on a hydrothermal method. Typically, MWNTs@SiO_2_ composite (230 mg) was dispersed into an ethanol (15 mL) and H_2_O (25 mL) mixture by sonification. Then, 1.2 g urea and 0.16 g K_2_SnO_3_·3H_2_O were dissolved in the above solution and sonicated for another 0.5 h. Subsequently, the suspension was transferred to a 50 mL Teflon‐lined stainless‐steel autoclave and kept at 170 °C for 36 h. To increase the amount of SnO_2_ loading, this process was repeated twice again.

*Synthesis of MWNTs@SiO_2_@SnO_2_@SiO_2_ Composite*: After SnO_2_ loading, 200 mg of obtained composite was dispersed into a mixture of 18 mL ethanol, 3 mL H_2_O, and 3 mL NH_3_.H_2_O and sonicated for 1 h. Then, 0.16 mL TEOS was added and stirred for 2 h. The product was washed with water and dried at 80 °C overnight.

*Synthesis of MWNTs@SnO_2_@C Composite*: The carbon coating was derived from the high temperature carbonization of RF resin. In a typical experiment, 400 mg MWNTs@SiO_2_@SnO_2_@SiO_2_ composite was dispersed in 28 mL H_2_O by ultrasonication, followed by addition of 0.1 mL NH_3_.H_2_O and 1 mL 0.01 m cetyltrimethylammonium bromide (CTAB) aqueous solution. After vigorously stirred for 0.5 h, 50 mg resorcinol and 0.07 mL formaldehyde solution were added and stirred for 16 h at room temperature. After washing with water and drying at 80 °C, the obtained powder was carbonized at 700 °C for 2 h under Ar atmosphere with a heating rate of 10 °C min^−1^. Finally, by etching the SiO_2_ layers with 2 m NaOH solution at 50 °C for 12 h, the MWNTs@SnO_2_@C composite with void space was obtained.

*Materials Characterization*: The morphology and structure of the samples were characterized via TEM (JEM‐2010F and FEI Tecani G2 F20), scanning electron microscopy (SEM, JSM‐7600F), X‐ray diffraction (Shimadzu, XRD‐6000), and Brunauer–Emmett–Teller surface area analyzer (BET, Micromeritics ASAP2020). TGA (Q500) were measured from 30 to 700 °C at a heating rate of 10 K min^−1^ in air.

*Electrochemical Measurements*: The electrochemical measurements of the samples were performed via CR2032 coin‐type test cells. The working electrodes were consisted of 80 wt% active materials (MWNTs@SnO_2_@C, or MWNTs@SnO_2_), 10 wt% conductivity agent (ketjen black, KB), and 10 wt% binder (carboxymethyl cellulose, Na‐CMC), which were mixed with deionized water and pasted on Ni foam. The electrodes were dried at 80 °C for 12 h in a vacuum before use. The cells were assembled in an Ar‐filled glove box. For LIBs, lithium foil was used as the counter electrode. Celgard 2300 membrane was the separator. The electrolyte was 1 m LiPF_6_ in ethylene carbonate (EC): ethylmethyl carbonate (EMC): dimethyl carbonate (DMC) (1:1:1 in volume). For NIBs, Na foil was the counter electrode and glass fiber (EL‐CELL) was the separator. The electrolyte was 1 m NaClO_4_ in ethylene carbonate (EC): diethyl carbonate (DEC) (1:1 in volume) with 10 wt% fluoroethylene. Cells were galvanostatically discharged and charged on a Neware Battery tester with current densities from 50 to 3200 mA g^−1^. Cyclic voltammetry (CV) test was carried out on a PINE WaveDriver 20 bipotentiostat with a scan rate of 0.5 mV s^−1^. For LIBs, the capacity values were calculated based on the total mass of the MWNTs@SnO_2_@C and MWNTs@SnO_2_ composite. For NIBs, the specific capacity was calculated based on the mass of SnO_2_, which is consistent with the calculation method established in NIB literatures.^15^

## Supporting information

As a service to our authors and readers, this journal provides supporting information supplied by the authors. Such materials are peer reviewed and may be re‐organized for online delivery, but are not copy‐edited or typeset. Technical support issues arising from supporting information (other than missing files) should be addressed to the authors.

Supplementary
