# Supplementary material for: Reserving Interior Void Space for Volume Change Accommodation: An Example of Cable‐Like MWNTs@SnO2@C Composite for Superior Lithium and Sodium Storage
Source: Adv Sci (Weinh). 2015 May 15;2(6):1500097. doi: 10.1002/advs.201500097 (PMC5029602; doi:10.1002/advs.201500097)
Supplement: Supplementary file 1 — Supplementary [file ADVS-2-0l-s001.pdf]

Copyright WILEY-VCH Verlag GmbH & Co. KGaA, 69469 Weinheim, Germany, 2015.

ADVANCED SCIENCE
Open Access

Supporting Information

for *Adv. Sci.*, DOI: 10.1002/advs.201500097

Reserving Interior Void Space for Volume Change Accommodation: An Example of Cable-Like MWNTs@ SnO_2@C Composite for Superior Lithium and Sodium Storage

*Yi Zhao, Chao Wei, Shengnan Sun, Luyuan Paul Wang, and Zhichuan J. Xu**

Copyright WILEY-VCH Verlag GmbH & Co. KGaA, 69469 Weinheim, Germany, 2013.

Supporting Information

Reserving interior void space for volume change accommodation: an example of cable-like MWNTs⊙SnO_2⊙C composite for superior lithium and sodium storage

*Yi Zhao, Chao Wei, Shengnan Sun, Luyuan Paul Wang, Zhichuan J. Xu**

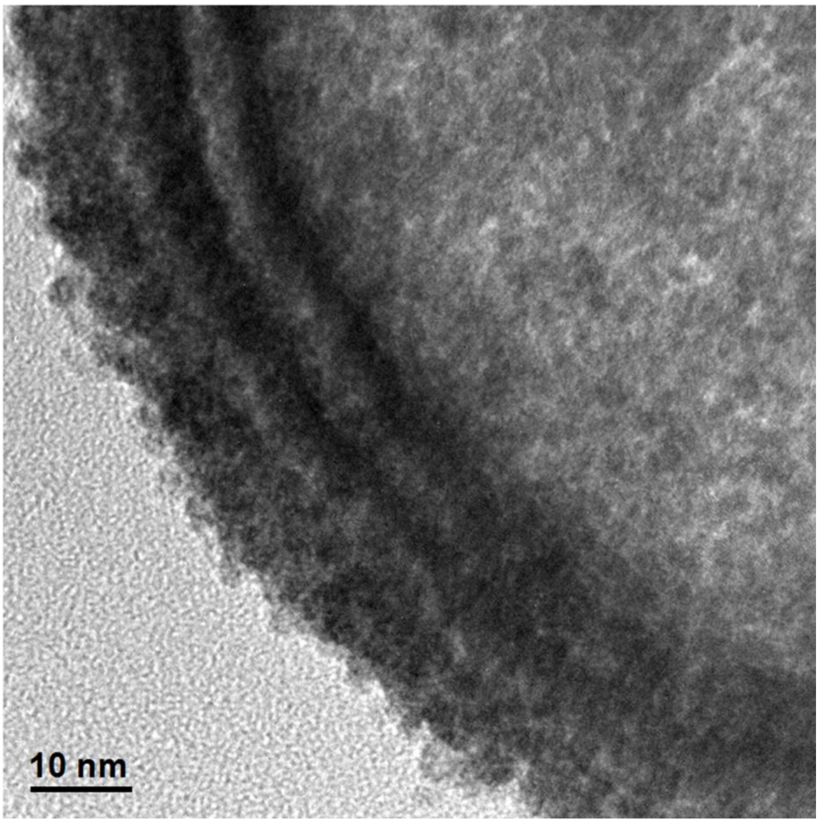

Figure S1. High magnification TEM image of MWNTs@SiO_2@SnO_2 composite, showing that SnO_2 layers were consisted with 2-3 nm nanoparticles.

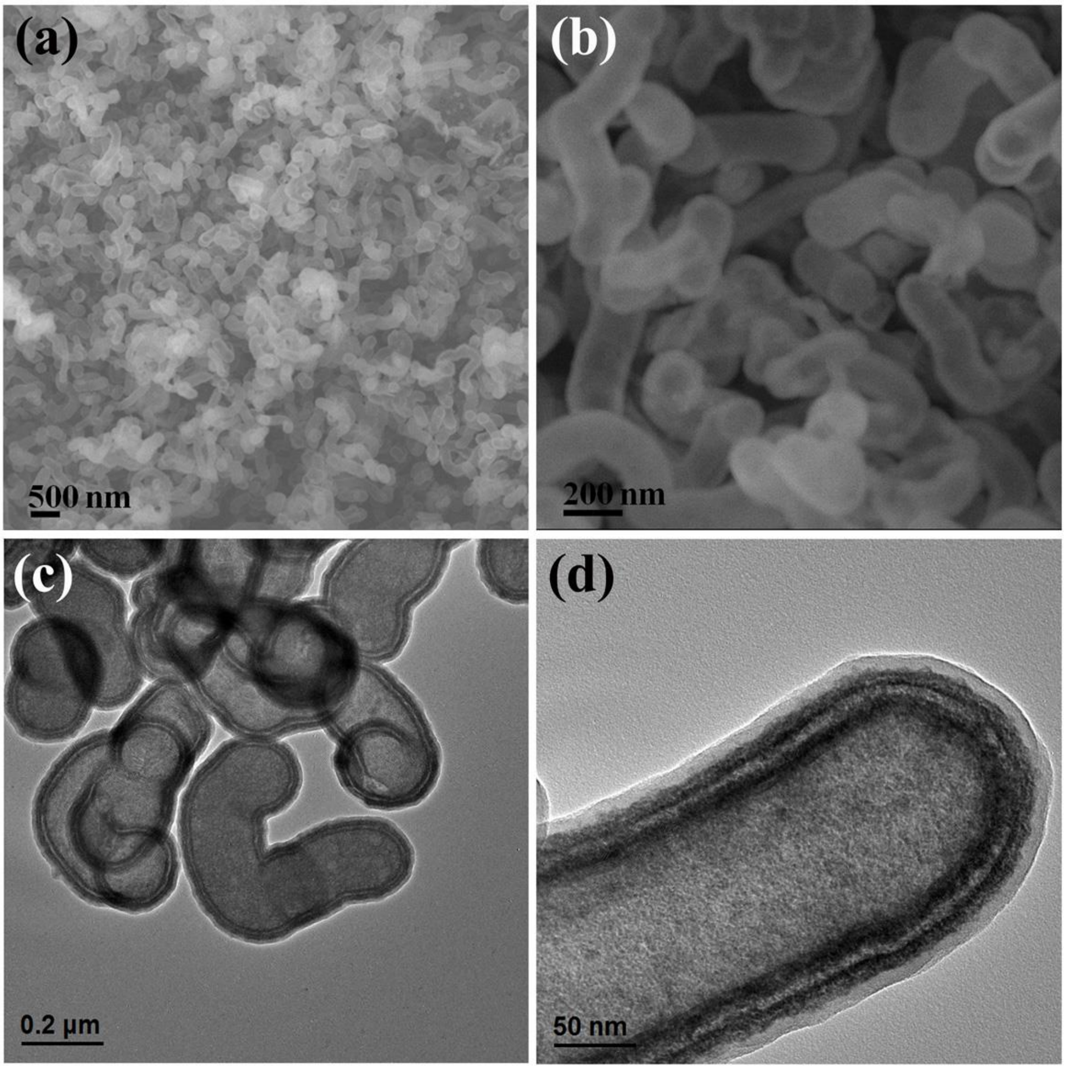

Figure S2. (a-b) SEM and (c-d) TEM images of MWNTs@SiO_2@SnO_2@SiO_2 composite. As can be seen, a thin SiO_2 layer (~8 nm) was uniformly coated on the surface of MWNTs@SiO_2@SnO_2 composite.

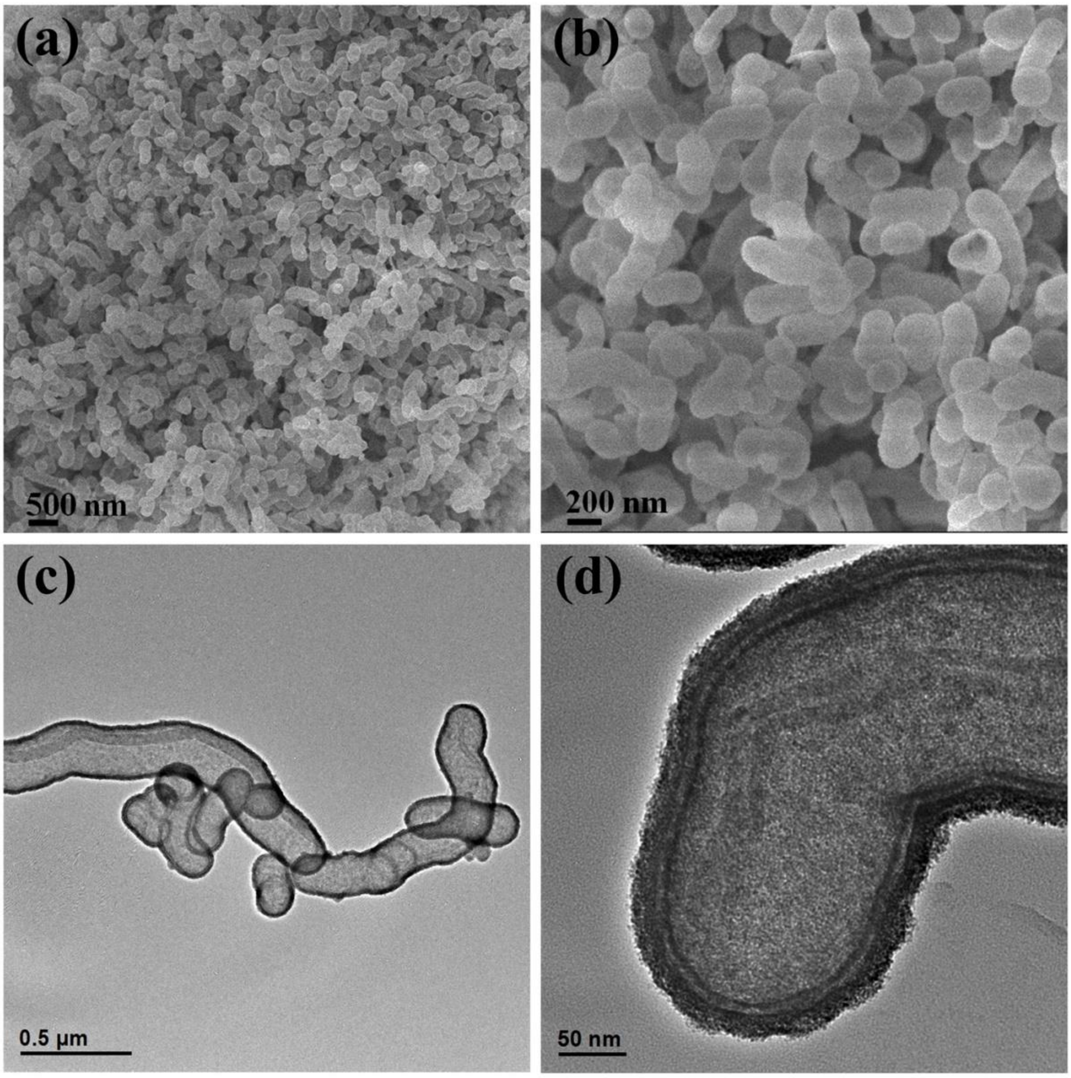

Figure S3. (a-b) SEM and (c-d) TEM images of MWNTs@SnO_2 composite, from which MWNTs were encapsulated within hollow SnO_2 layers with interior void space.

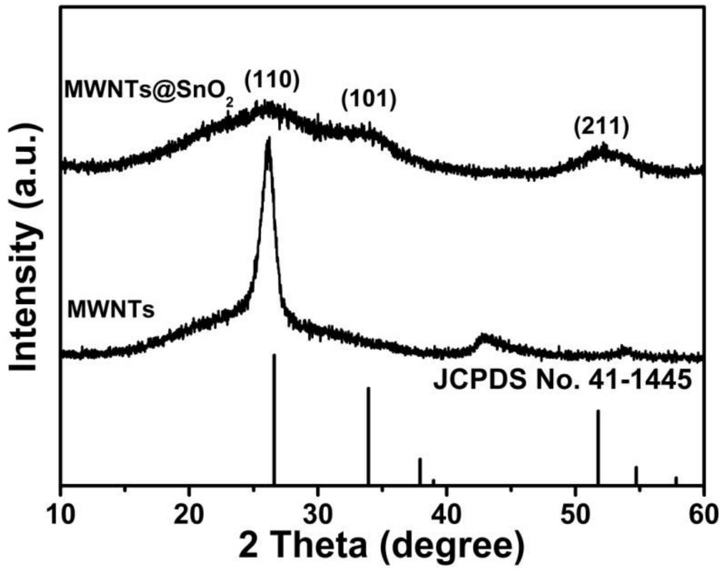

Figure S4. XRD patterns of MWNTs and MWNTs@SnO_2 composite. The main diffraction peaks of MWNTs@SnO_2 can be well assigned to the tetragonal structure of SnO_2 (JCPDS No. 41-1445). The broad diffraction peaks of MWNTs@SnO_2 indicated the small particle size of SnO_2 in this composite. Meanwhile, the diffraction peak of MWNTs around 26° was overlapped with the (110) planes of SnO_2.

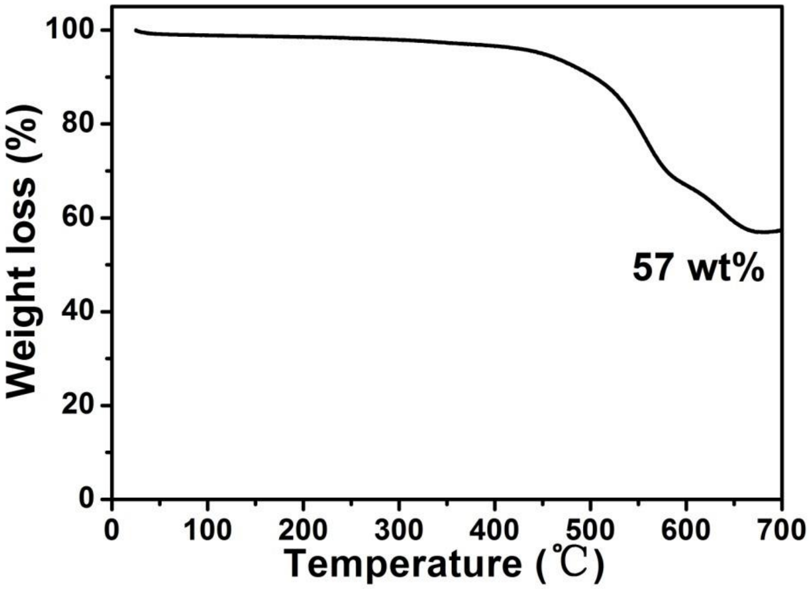

Figure S5. Thermogravimetry analysis (TGA) curve of MWNTs⊙SnO_2⊙C composite at a heating rate of 10 K min^{-1} between 30 and 700 ℃ in air. As can be seen, this TGA curve exhibited two distinct weight loss regions at 400-580 ℃ and 580-670 ℃, corresponding to the removal of carbon layer and MWNTs, respectively. The final SnO_2 content in this composite was 57 wt%.

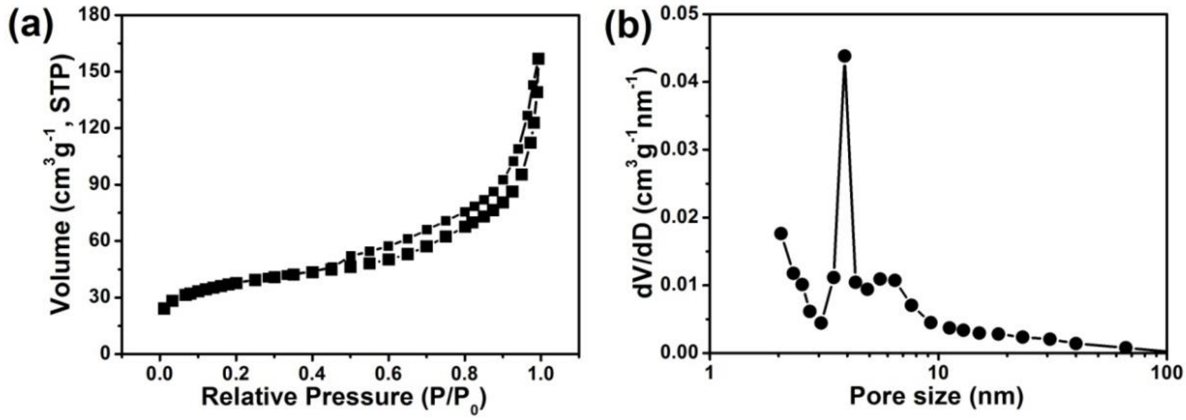

Figure S6. (a) Nitrogen adsorption—desorption isotherms and (b) pore size distribution of the MWNTs@SnO_2 composite, which had a BET surface area of 129 m^2 g^{-1} and total pore volume of 0.23 m^3 g^{-1}.

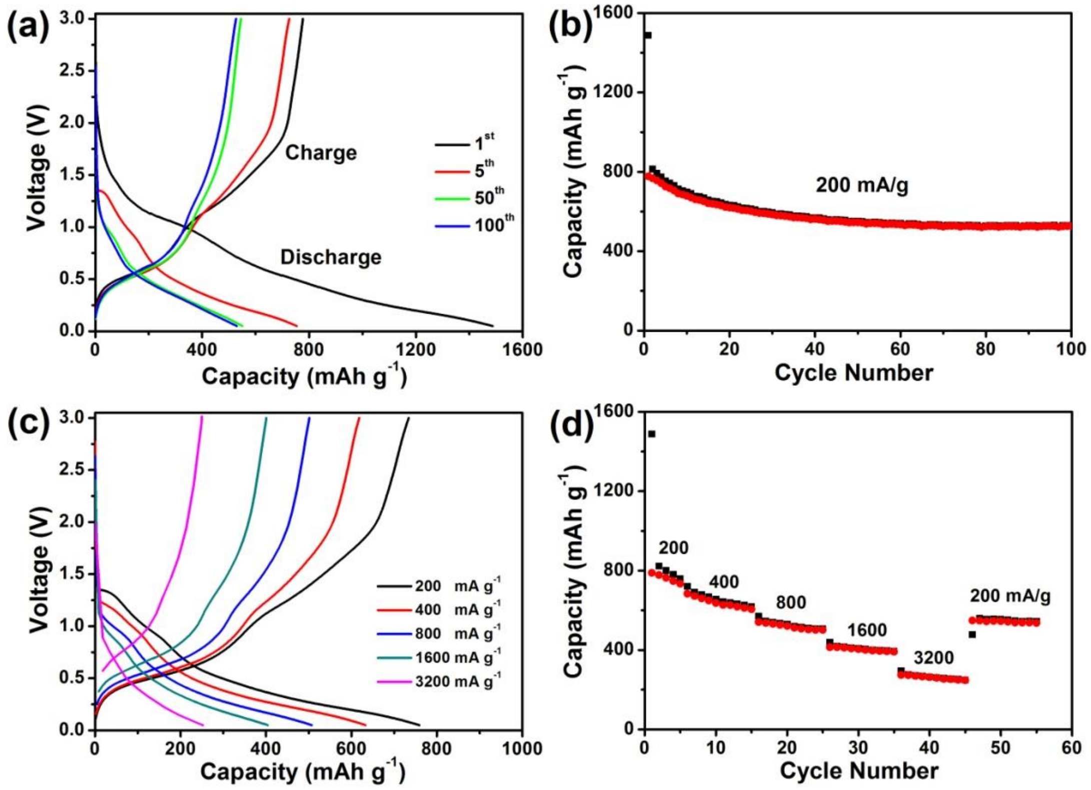

Figure S7. Electrochemical properties of MWNTs@SnO_2 electrode between 0.05 V and 3.0 V for LIBs. (a) Discharge/charge curves, and (b) cycling performance at 200 mA g^{-1}. (c) Typical discharge and charge profiles, and (d) rate capabilities at various current densities from 200 to 3200 mA g^{-1}. This MWNTs@SnO_2 composite without carbon layer protection showed an initial columbic efficiency of 52.2 % and suffered from gradual capacity fading with only only 527 mAh g^{-1} remained after 100 cycles. Meanwhile, this electrode also exhibited a poor rate performance and only showed a low capacity of 250 mAh g^{-1} at 3200 mA g^{-1}.

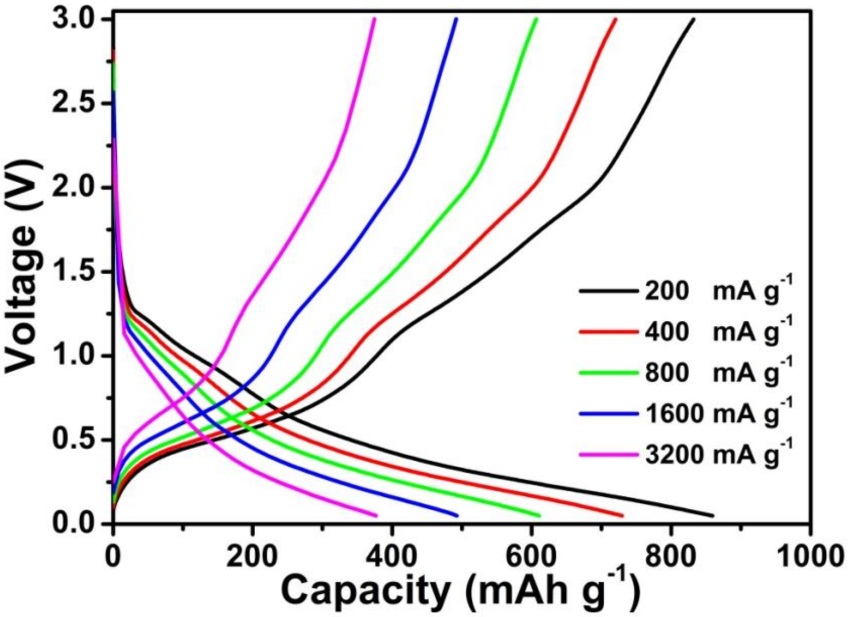

Figure S8. Typical discharge and charge curves of the MWNTs⊙SnO_2⊙C electrode at various rates from 200 to 3200 mA g^{-1}. Even at large current densities, these curves still remained the reaction plateaus and delivered high reversible capacities.

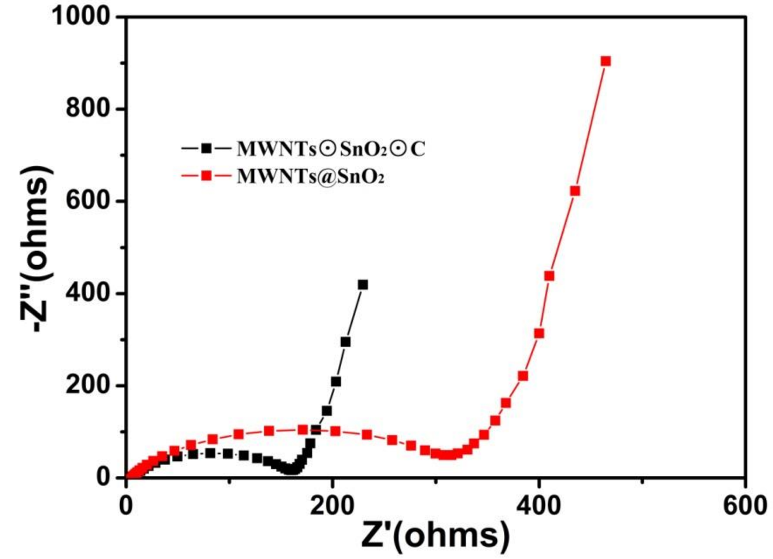

Figure S9. Electrochemical impedance spectra for MWNTs⊙SnO_2⊙C and MWNTs@SnO_2 electrodes.

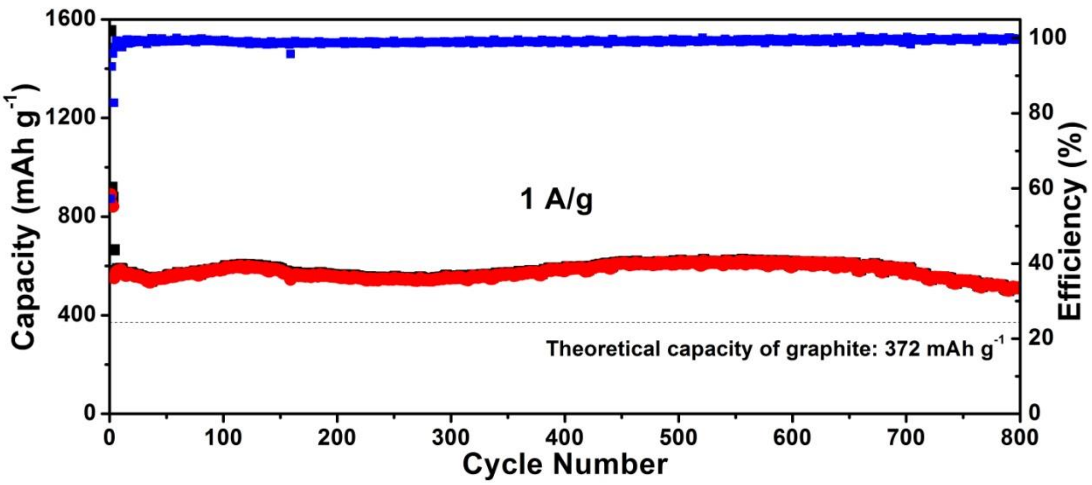

Figure S10. Long-term cycling performance of MWNTs⊙SnO_2⊙C electrode at a high rate of 1 A g^{-1} after being activated at 200 mA g^{-1} in the initial three cycles. As can be seen, this electrode also delivered outstanding cycling performance at 1 A g^{-1}, which preserved high specific capacities of 615 and 513 mAh g^{-1} after 500 and 800 cycles, respectively.

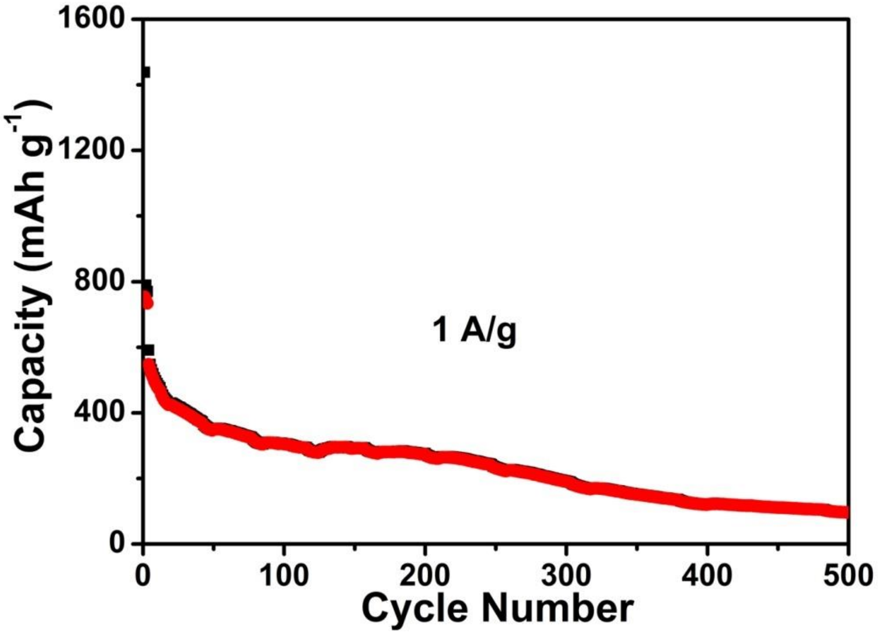

Figure S11. Cycling performance of the MWNTs@SnO_2 electrode at a large current density of 1 A g^{-1}. The MWNTs@SnO_2 electrode showed a poor cycling performance at a high current densities of 1 A g^{-1} with a low capacity of 96 mAh g^{-1} remained after 500 cycles.

Table S1. Electrochemical performance of the MWNTs@SnO_2 based composites for LIBs.

Materials	Current density (mA g^{-1})	Cycle Number	Capacity (mAh g^{-1})	Reference
MWNTs@SnO_2	33.3	50	344.5	[1]
MWNTs@SnO_2	78	20	404	[2]
MWNTs@SnO_2	200	40	362	[3]
MWNTs@SnO_2	78	45	464	[4]
MWNTs@SnO_2	100	100	473	[5]
MWNTs@SnO_2	200	300	497	[6]
MWNTs@SnO_2 nanosheets	160	40	549	[7]
MWNTs@SnO_2@Au	180	40	626	[8]
MWNTs@SnO_2@C	100	65	462	[9]
MWNTs@SnO_2@C	400	60	505	[10]
MWNTs@SnO_2 nanorods@C	720	40	698	[11]
MWNTs@SnO_2@PPy	100	30	600	[12]
**MWNTs⊙SnO_2⊙C**	200<br>1000<br>1600	100<br>800<br>1300	944<br>513<br>412	**this work**

[1] Z. H. Wen, Q. Wang, Q. Zhang and J. H. Li, *Adv. Funct. Mater.* **2007**, *17*, 2772.
[2] Z. Y. Wang, G. Chen and D. G. Xia, *J. Power Sources* **2008**, *184*, 432.
[3] C. H. Xu, J. Sun and L. Gao, *J. Phys. Chem. C* **2009**, *113*, 20509.
[4] L. S. Zhang, L. Y. Jiang, C. Q. Chen, W. Li, W. G. Song and Y. G. Guo, *Chem. Mater.* **2010**, *22*, 414.
[5] L. Noerochim, J. Z. Wang, S. L. Chou, H. J. Li and H. K. Liu, *Electrochim. Acta.* **2010**, *56*, 314.
[6] J. G. Ren, J. B. Yang, A. Abouimrane, D. P. Wang and K. Amine, *J. Power Sources* **2011**, *196*, 8701.
[7] S. J. Ding, J. S. Chen and X. W. Lou, *Adv. Funct. Mater.* **2011**, *21*, 4120.
[8] G. Chen, Z. Wang and D. Xia, *Chem. Mater.* **2008**, *20*, 6951.
[9] P. Wu, N. Du, H. Zhang, J. X. Yu and D. R. Yang, *J. Phys. Chem. C* **2010**, *114*, 22535.
[10] S. J. Ding, J. S. Chen and X. W. Lou, *Chem. Asian J.* **2011**, *6*, 2278.
[11] S. Chen, Y. L. Xin, Y. Y. Zhou, F. Zhang, Y. R. Ma, H. H. Zhou and L. M. Qi, *J. Mater. Chem. A* **2014**, *2*, 15582.
[12] Q. G. Shao, W. M. Chen, Z. H. Wang, L. Qie, L. X. Yuan, W. X. Zhang, X. L. Hu and Y. H. Huang, *Electrochem. Commun.* **2011**, *13*, 1431.

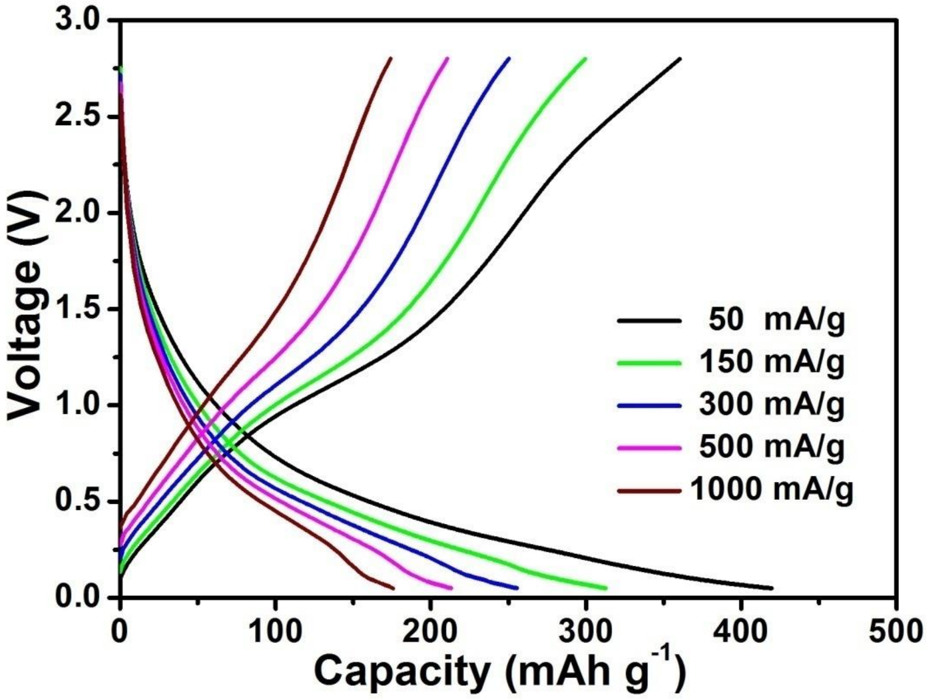

Figure S12. Typical discharge/charge profiles at various current densities from 50 to 1000 mA g^{-1} of the MWNTs⊙SnO_2⊙C electrode for NIBs.

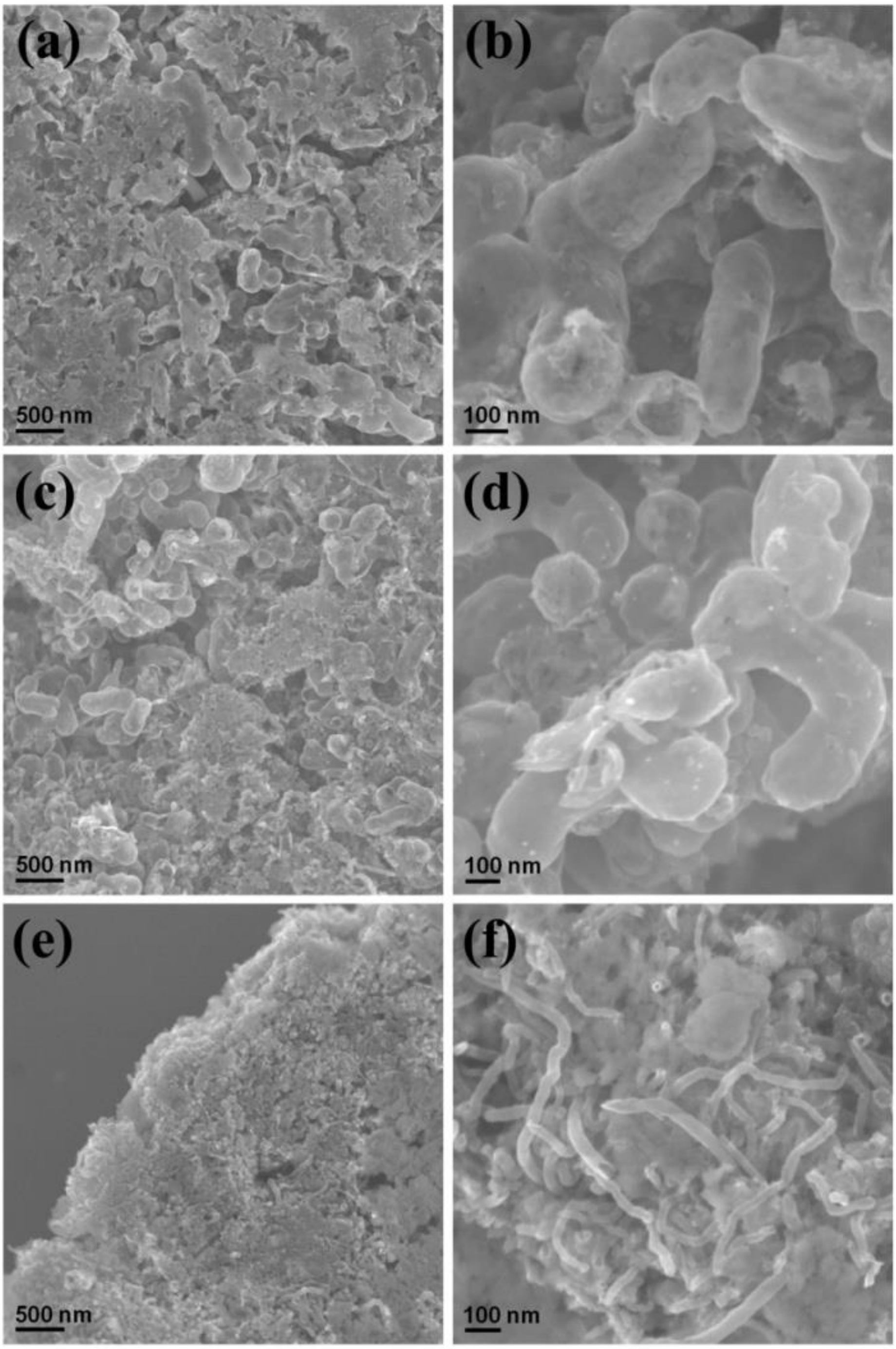

Figure S13. SEM images of MWNTs⊙SnO_2⊙C electrode after (a-b) lithium and (c-d) sodium insertion/extraction cycles. (e-f) SEM images of MWNTs@SnO_2 electrode after lithium storage cycles. Seen from the above SEM images, the MWNTs⊙SnO_2⊙C composite was able to preserve the original one-dimensional morphology after several lithium or sodium storage cycles, confirming the good structure stability of this architecture. While, the MWNTs@SnO_2 suffered from structure pulverization during lithium storage process with only MWNTs observed after several cycles.
